# Supplementary material for: Early Changes in Circulating FGF19 and Ang-2 Levels as Possible Predictive Biomarkers of Clinical Response to Lenvatinib Therapy in Hepatocellular Carcinoma
Source: Cancers (Basel). 2020 Jan 26;12(2):293. doi: 10.3390/cancers12020293 (PMC7073176; doi:10.3390/cancers12020293)
Supplement: Supplementary file 1 [file cancers-12-00293-s001.pdf]

Supplementary Materials: Early Changes in Circulating FGF19 and Ang-2 Levels as Possible Predictive Biomarkers of Clinical Response to Lenvatinib Therapy in Hepatocellular Carcinoma

Makoto Chuma, Haruki Uojima, Kazushi Numata, Hisashi Hidaka, Hidenori Toyoda, Atsushi Hiraoka, Toshifumi Tada, Shunji Hirose, Masanori Atsukawa, Norio Itokawa, Taeang Arai, Makoto Kako, Takahide Nakazawa, Naohisa Wada, Shuitirou Iwasaki, Yuki Miura, Satoshi Hishiki, Shuhei Nishigori, Manabu Morimoto, Nobuhiro Hattori, Katsuaki Ogushi, Akito Nozaki, Hiroyuki Fukuda, Tatehiro Kagawa, Kojiro Michitaka, Takashi Kumada and Shin Maeda

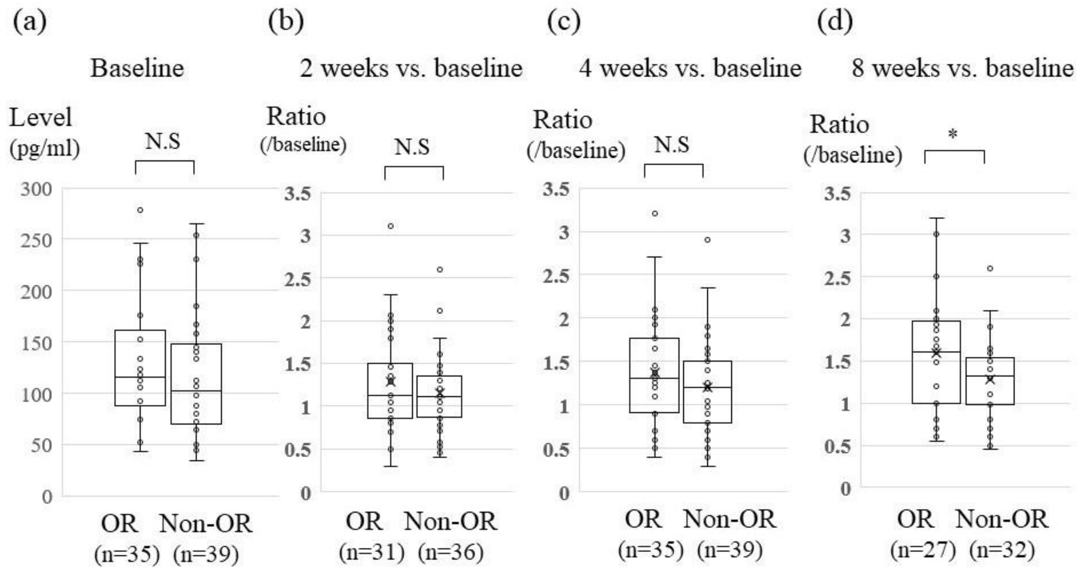

Figure S1. Association between serum FGF 23 levels and lenvatib treatment response. Distribution of serum FGF23 levels at baseline (**a**) and changes in FGF23 levels from baseline at 2 weeks (**b**), 4 weeks (**c**), and 8 weeks (**d**) between the OR (Objective response) and non-OR groups. Data are shown as median values (10th-90th percentile ranges). * $p < 0.05$, N.S: non-significant.

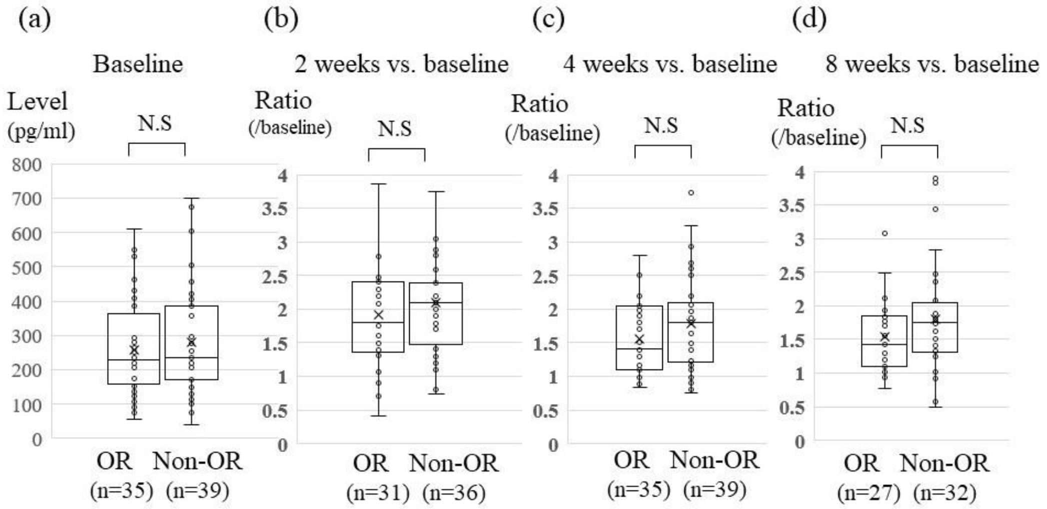

Figure S2. Association between serum VEGF levels and lenvatinib treatment response. Distribution of serum VEGF levels at baseline (**a**) and changes in VEGF levels from baseline at 2 weeks (**b**), 4 weeks

(**c**), and 8 weeks (**d**) between the OR (Objective response) and non-OR groups. Data are shown as median values (10th-90th percentile ranges). The Mann-Whitney or Kruskal-Wallis test was used to determine statistical significance. N.S: non-significant.

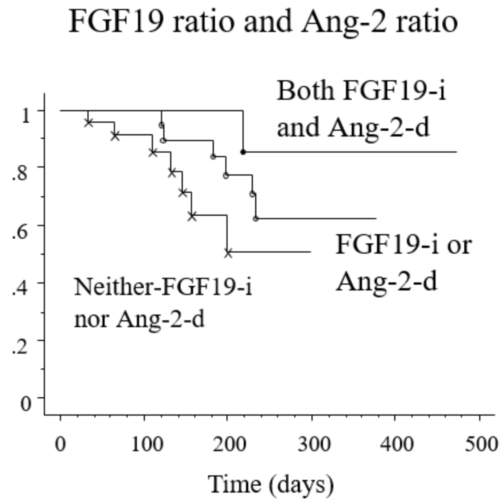

Figure S3. Association between changes in FGF19 and Ang-2 levels and overall survival. Kaplan-Meier analysis of 74 hepatocellular carcinoma (HCC) patients who received lenvatinib treatment, stratified according to ratio of FGF19 and Ang-2. FGF19-i represents a FGF19 ratio at 4 weeks/baseline of ≥1.51 and Ang-2-d represents an Ang-2 ratio at 4 weeks/baseline of ≤0.67.

© 2020 by the authors. Licensee MDPI, Basel, Switzerland. This article is an open access article distributed under the terms and conditions of the Creative Commons Attribution (CC BY) license (http://creativecommons.org/licenses/by/4.0/).
